# A New Approach to Monitoring Dengue Activity

**DOI:** 10.1371/journal.pntd.0001215

**Published:** 2011-05-31

**Authors:** Lawrence C. Madoff, David N. Fisman, Taha Kass-Hout

**Affiliations:** 1 Division of Infectious Disease and Immunology, University of Massachusetts Medical School, Worcester, Massachusetts, United States of America; 2 Division of Epidemiology and Immunization, Massachusetts Department of Public Health, Boston, Massachusetts, United States of America; 3 Dalla Lana School of Public Health, University of Toronto, Toronto, Ontario, Canada; 4 Departments of Health Policy, Management and Evaluation, University of Toronto, Toronto, Ontario, Canada; 5 Department of Medicine, University of Toronto, Toronto, Ontario, Canada; 6 Public Health Surveillance Program Office, Office of Surveillance, Epidemiology & Laboratory Services, U.S. Centers for Disease Control and Prevention, Atlanta, Georgia, United States of America; Yale School of Public Health, United States of America

Among the world's most vexing emerging infectious diseases, dengue continues to spread, and in its many endemic areas is a major public health problem [1]–[3]. There is no vaccine available, and the immunology of dengue, whereby immunological “priming” can result in extremely severe manifestations (e.g., dengue hemorrhagic fever) complicates vaccine development [4]. Thus dengue control is dependent on controlling the mosquito vector, and resistance to insecticides, environmental and social disruption, climate change, and global movement of goods and people (and incidentally, vectors) provide ongoing hurdles to effective vector control [5], [6].

Accurate risk analysis and allocation of resources for dengue control depends on disease surveillance. Dengue surveillance is similarly complex and depends in most areas on formal surveillance systems that capture case counts (or via syndromic surveillance at sentinel sites) [7], [8]. Laboratory reporting of serology can confirm not only individual cases but identify the viral serotypes found in a given area at a point in time [9]. Analysis of mosquito populations can also confirm dengue circulation and provide information on viral types [10]–[12]. Formal surveillance has many advantages: precise counts of case numbers, good geographic localization and the potential to identify precise disease etiology among them.

However, formal infectious disease surveillance systems have important limitations, including lags between case occurrence and reporting. Sentinel sites may report cases only periodically or fail to report altogether for a variety of reasons. Delays in reporting may occur when governmental organizations charged with surveillance aren't able to adequately collect and analyze data or publish reports in a timely manner. These problems may be particularly daunting in developing countries with limited resources to devote to strengthening surveillance systems: robust formal public health surveillance is expensive, requiring major investments in trained personnel, communications, buildings and equipment. Indeed, the economic conditions that prevent development of robust surveillance systems may also be those that potentiate dengue transmission: for example, a seroprevalence study performed in a city straddling the Texas-Mexico border found marked differences in dengue seroprevalence on the Mexican side of the border in association with economic disadvantage [13].

The hierarchical nature of formal public health surveillance also poses challenges to surveillance. Hierarchical reporting structures can lose data at any point of interaction, for example when a regional authority fails to report to a national one. Finally, in some situations there can be short-term disincentives for the timely and transparent reporting of disease activity: governments may fear that surges in disease activity may chase away tourists or visitors, or may undermine government credibility [14].

To address these drawbacks, a complementary system of informal surveillance tools have been developed, some by governmental agencies, but many by non-governmental organizations and/or researchers. Event-based surveillance systems such as ProMED, GPHIN HealthMap and BioCaster rely on unofficial reports of disease, for example from clinicians or web-based healt-related news media, to report on disease outbreaks [15]–[17]. Such systems have proven reliable and timely and informal sources of information were even recognized in the 2005 revision of the International Health Regulations as important sources of epidemic intelligence [18], [19]. The rapid and accelerating growth of the Internet has improved the usefulness and sensitivity of these systems and they have likely improved the timeliness of outbreak reporting [18], and the ever-expanding availability of electronic information has also led to the discovery of other types of analyses that detect disease outbreaks. “Web-crawlers” (software programs that search internet sites for specific terms, and then use these search terms to generate reports or maps of disease activity) can provide important information on disease outbreaks that may be published on nongovernmental websites, in online newspapers, and in blogs, and this approach powers the widely-used HealthMap system mentioned above [20]. In the context of the recent cholera outbreak in Haiti, there were inconsistencies in initial accounts of regional disease activity, but information from HealthMap proved useful in the construction of a mathematical model that predicted disease spread on the island [21].

The analysis of real time search queries—the so-called “searchstream”– has been shown to be a sensitive and timely means of evaluating geographically-specific trends in influenza; both Yahoo and Google search engines have proven to be powerful tools for influenza surveillance [22], [23]. More recently, evaluation of data from the microblogging website Twitter has been shown to provide useful information about *both* disease activity and disease concern related to the 2009 influenza pandemic [15]. Finally, the widespread availability of smartphone technologies makes it possible to interact with population members to elicit information on illness (so-called “crowdsourcing”), and also (by using cellphone or smartphone network data) to evaluate the movement of populations, which may be a key predictor of how epidemics spread [24]–[26].

**Figure 1:**
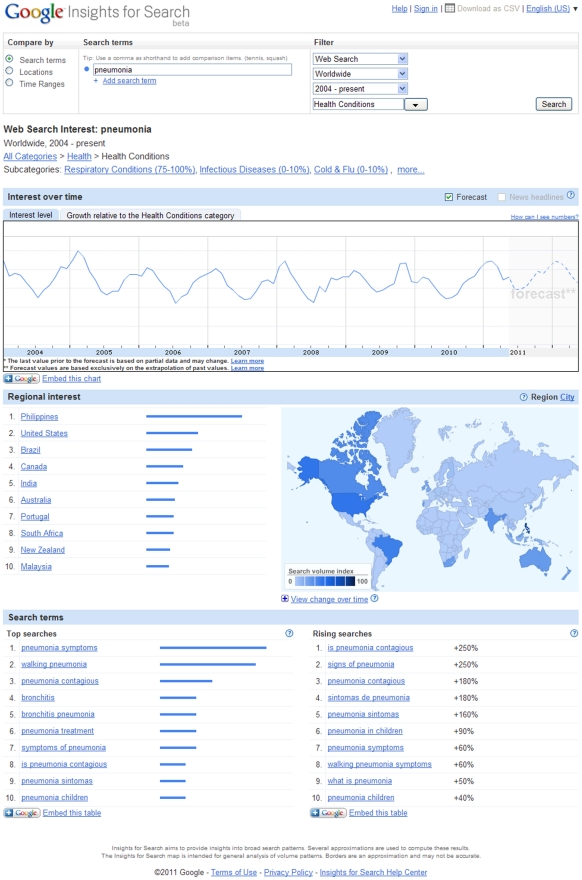
Screenshot of search performed on the term “pneumonia” using the Google Insights for Search tool (http://www.google.com/insights/search/#). The expected wintertime seasonality of pneumonia incidence is mirrored in seasonal surges in search volumes.

**Figure 2:**
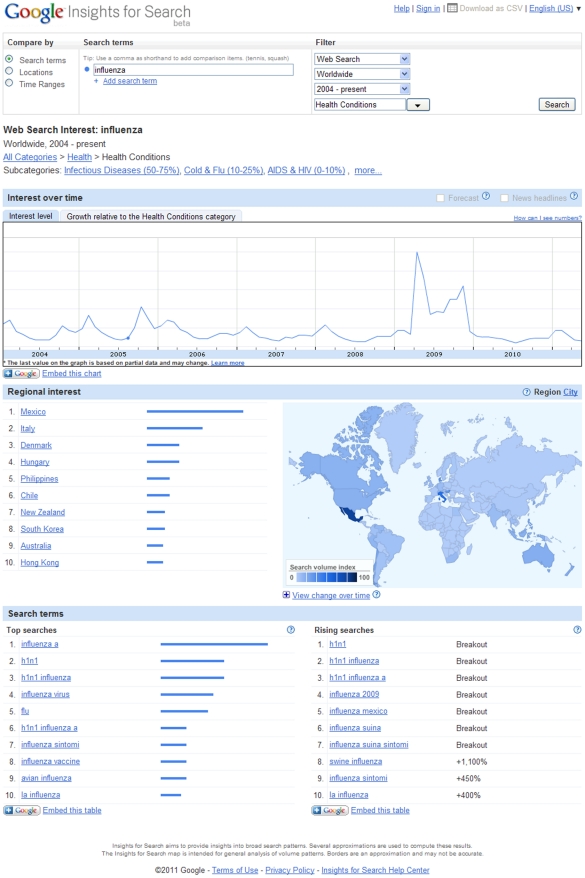
Screenshot of search performed on the term “influenza” using the Google Insights for Search tool (http://www.google.com/insights/search/#). Although influenza searches would expect to display similar wintertime seasonality to pneumonia searches, depicted in Figure 1, the public concern and interest generated by the 2009 influenza pandemic generated a large spike in searches in that year, which obscures seasonal oscillation in other years.

Readers unfamiliar with these approaches may wish to try a simple experiment using the Google Insights for Search tool, which provides a graphical depiction of both search term volumes and online media reports of disease (http://www.google.com/insights/search/#). Searches on terms such as “norovirus” or “pneumonia” produce seasonally oscillating patterns of searches as one might anticipate in diseases with strong wintertime seasonality (Figure 1), and which is presumably generated by individuals who have, or know someone who has, this diagnosis seeking to learn more about it online. However, the pitfalls of this approach can be appreciated in a similar manner: a search on the term “influenza” produces a graph with a tremendous spike in 2009 (Figure 2); indeed a spike so large that it obscures influenza activity in all other years. This reflects the difficulties that searchstream-based surveillance methods may encounter when evaluating diseases that generate extreme public concern or media attention.

Chan et al., in this edition of *PLoS Neglected Tropical Diseases* [27], apply searchstream surveillance techniques to the monitoring of dengue. In this case, search queries appear to closely track (rather than lead) dengue activity as measured by traditional systems. The authors have limited their model to certain locations defined in part by the extent of Internet use in these areas (Bolivia, Brazil, India, Indonesia and Singapore). Their findings are exciting: when evaluated in a “testing set” of data not used to derive initial models, they found extremely strong correlation between dengue-related query volumes and case counts reported by traditional surveillance systems, but their approach has the advantage of both timeliness and transparency (including the availability of the system on the Google.org website).

As with any prediction-oriented surveillance tool, a major concern relates to model “over-fitting” such that the prediction model performs well in the dataset that was used in its creation but fails to work well in the “real world”. Reassuringly the authors divided their data into a derivation set and a testing set (or “holdout set” as they call it), with the former used for model construction. As can be seen in the table and figure they present, their derived models perform extremely well in both sets in all countries, in the derivation set as expected, but also in the testing set. Perhaps less straightforward is the authors' decision to “smooth out” unusual spikes in search volumes in candidate queries; as demonstrated by the influenza example above, extreme surges in public interest in a disease can cause surges in query volumes, as can surges in interest related particular subject that is unrelated to the disease under surveillance but shares attributes that would be the subject of searches. By smoothing search volumes, the authors may have incorporated into their models terms that have the potential to “misbehave” in the future. For example, one imagines that if a novel (and frightening) new hemorrhagic fever unrelated to dengue emerges in one of these countries in coming years, one would imagine that the correlation between the search term “haemorrhagic fever” and dengue volumes would decline. As we don't have access to the precise query terms that were included in each country-specific model, it is difficult to know whether or not the terms included in the model would be vulnerable to such effects. The authors note that the expanding range of a clinically similar illness (Chikungunya) may confound the utility as well [28].

It would also be helpful to see to what extent there is overlap in components of models across countries, as this may help us understand whether these models can be applied to other jurisdictions or whether they are applicable only in the country for which they were constructed. As dengue is a disease whose range may change under the influence of climate change, it is important to know whether such an approach is applicable in the face of novel emergence of dengue in a new region or jurisdiction, or whether it is only applicable in countries like these in which dengue is currently endemic.

Perhaps the greatest challenge for the use of the approach described here is the same that applies across surveillance modalities: the same geographic locations that lack public health resources to control dengue, and to perform traditional surveillance, are likely to lag in access to the Internet as well. Nonetheless, the application of web-query based monitoring to a major and growing health threat in the developing world represents an important step forward. The ability to inexpensively and reliably maintain situational awareness of dengue activity will be welcomed by those charged with the public health response.

Does the development of web-based surveillance tools represent a revolution in how we conceptualize surveillance? We think not: current high-quality public health surveillance already utilizes multiple sources of information to gain a more complete picture of the incidence and distribution of disease. For example, influenza surveillance may include laboratory-based virological surveillance, sentinel syndromic surveillance (e.g., school-based absenteeism reports) and evaluation of mortality trends for pneumonia and influenza, which taken together may provide a more complete picture of disease risk and impacts. Searchterm-based surveillance and other modalities mentioned above thus provide an additional tool in the surveillance toolbox, which has advantages over traditional surveillance as well as limitations. It should be noted, however, that limitations such as those described above are not absent from traditional surveillance systems either: estimates of incidence can change markedly with changing case definitions, incidence of laboratory-confirmed disease can change markedly with augmentation or restriction of clinical testing or changes in diagnostic test methodologies, and syndromic surveillance systems can be subject to poor specificity and frequent false alarms. Thus supplementary information derived using methods such as the one developed by Chan and colleagues should be welcomed by public health professionals. The transparency of such systems may also help demonstrate the value of openness in disease reporting, which may have “spillover effects” on traditional surveilance systems.
